# The Effect of Deworming Using Triple-Dose Albendazole on Nutritional Status of Children in Perobatang Village, Southwest Sumba, Indonesia

**DOI:** 10.1155/2017/5476739

**Published:** 2017-11-08

**Authors:** Saleha Sungkar, Asri S. Ridwan, Gladys Kusumowidagdo

**Affiliations:** Department of Parasitology, Faculty of Medicine, Universitas Indonesia, Jakarta, Indonesia

## Abstract

High prevalence of STH leads to malnutrition, anemia, cognitive impairment, and growth disorders. Triple-dose albendazole 400 mg is a broad-spectrum anthelminthic; however, its effectiveness varies in every region. This study aims to determine the benefits of deworming using triple-dose albendazole on children's nutritional status in Perobatang Village, Southwest Sumba, Indonesia. This pre-post study was conducted in July 2016 and January 2017. Children aged 1–15 years were asked to collect stool for diagnosis of STH infection (Kato-Katz method), were measured for anthropometry status to obtain the nutritional status, and took albendazole 400 mg for three consecutive days. Data was analyzed with SPSS version 20. Prevalence of STH prior to the treatment was 95.4%:* T. trichiura* 85.2%,* A. lumbricoides* 71.6%, and hookworm 18.2%. After treatment, prevalence of STH decreased significantly (McNemar test, *p* < 0.001) to 53.4%, (*T. trichiura* 39.8%,* A. lumbricoides* 22.7%, and hookworm 1.1%). Before treatment, 33% participants were in normal nutritional status, 47.7% underweight, and 19.3% severely underweight. After treatment, children in normal nutritional status increased to 75%, underweight children decreased to 25%, and there were no severely underweight children. In conclusion, deworming with triple-dose albendazole 400 mg is effective in improving the nutritional status of children in Perobatang Village.

## 1. Background

Soil transmitted helminths (STH) are worms whose life cycle requires soil for its embryonic maturation process. The most common STH infecting humans are* A. lumbricoides*,* T. trichiura*, and hookworm* (A. duodenale and N. americanus)* [1–3].

In Indonesia, especially in areas with poor hygiene, sanitation, and low socioeconomic status, the prevalence of STH is still high. In 2013, the prevalence of STH infection in various districts of Indonesia was more than 20%, one of which is located in Southwest Sumba District (SSD) [4, 5]. Perobatang Village is an underdeveloped area in SSD which has difficulty in obtaining clean water; thus, water is used for drinking and cooking only, rarely for hand washing before eating and after defecating. This condition is worsened by the practice of open defecation, a risk factor for STH infection [6, 7].

STH could infect people in all ages but more often in children due to poor personal hygiene.* A. lumbricoides* and* T. trichiura* affect the process of digestion, absorption, and food metabolism, whereas hookworms cause chronic intestinal blood loss that result in anemia. Therefore, STH could cause physical and cognitive developmental disorders and decrease body immunity, making it susceptible to other diseases [8, 9]. Hence, deworming should be done in areas with high risk of STH.

STH control could resolve anemia and in turn improve children's nutritional status [10]. According to WHO, if a sampling area shows STH prevalence is more than 50%, mass treatment should be conducted every 6 months and once a year when prevalence is 20–50%, and if the prevalence is less than 20%, selective treatment is carried out using single dose of 400 mg albendazole [11].

Single-dose albendazole is effective in controlling* A. lumbricoides* and hookworm but not* T. trichiura*. For treatment of* T. trichiura*, triple-dose albendazole gave higher cure rates (56%) than single dose (31%) [9]. However, the cure rates of albendazole vary geographically. Based on that fact, it is necessary to conduct a study to investigate the benefits of triple-dose albendazole in reducing STH prevalence and its effect on improving the nutritional status of children in Perobatang Village, SSD. The village was chosen due to high risk of STH infection and there was no history of previous mass drug administration with albendazole in the village.

## 2. Methods and Materials

**Figure 1:**
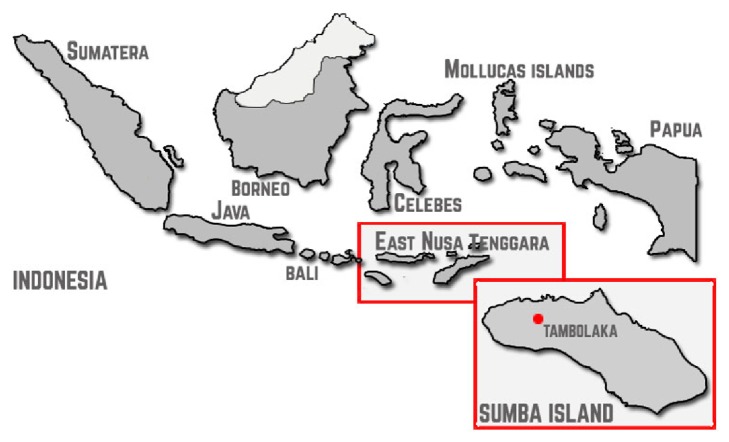
Geographical map of study location. Tambolaka is the capital of SSD, Indonesia, and Perobatang Village is a 2-hour drive from the capital.

This study used a pre-post study design and was conducted in Perobatang Village, SSD, Indonesia (Figure 1). Data was taken in July 2016 (pretest) and January 2017 (posttest). All children aged 1–15 years who were willing (having parental permissions) to follow the study and did not take anthelmintic in the last six months were included. Severely ill or febrile children were excluded. The dropout criteria were participants who did not collect the fecal samples or did not present during the posttest.

### 2.1. Procedures

All children in the village were included in the study. The data was collected by performing anthropometry assessment and fecal examination. Data retrieval was done twice at pretest and posttest with six-month interval.

On the first day, participants were given explanation regarding the study and asked for informed consent. To obtain the nutritional status, anthropometry measurement was done by measuring height and weight. The participants were asked to remove any hat, caps, and footwear during height measurement to gather exact measurement. Digital scale, prior to calibrated, was used to measure the weight. After the examination was completed, the participants were given explanation on how to collect the fecal samples. Thumb-sized feces were put into 10 cc pot which had been labeled. On the next day, the pot containing feces was returned to the researcher for Kato-Katz method preparations. The samples were examined by light microscope to identify eggs or larvae of the worms. Kato-Katz method was chosen to count the eggs to determine the intensity of infections. The egg counts were later used to determine the intensity of infection (see Supplementary Table 1 in Supplementary Material available online at https://doi.org/10.1155/2017/5476739).

Participants were treated with albendazole 400 mg (2–15 years) and 200 mg (1-2 years) for three consecutive days and the administration of the drugs was witnessed by the researchers. Triple-dose albendazole was administered rather than single dose due to its higher cure rate for* T. trichiura* [9]. To increase the absorption of albendazole, the drugs were taken with milk and milk biscuits.

The data was analyzed using McNemar test in SPSS version 20 to identify the association between the prevalence of STH before and after the treatment and Wilcoxon test for the nutritional status. Deworming is considered to be successful if there is a significant difference in the decrease of the prevalence of STH and the increase of nutritional status before and after deworming. The value of *α* was 0.05 and confidence interval (CI) was 95%. *p* value of less than 0.05 is considered significant.

Ethical approval was obtained from the ethics committee Faculty of Medicine Universitas Indonesia number 876/UN2.F1/ETIK/2016.

## 3. Results

### 3.1. The Prevalence of STH before and after Deworming

From 192 children aged 1–15 years in Perobatang Village, 109 children were registered as participants of this study; however, 88 participants were included in the analysis due to exclusion and dropout criteria.

Based on the WHO chart of weight-for-age and BMI-for-age, the category of children was divided into two: 1–5 years and 6–15 years. A total of 31 children (35.2%) aged 1–5 years and 57 children (64.8%) aged 6–15 years were included in this study. The prevalence of STH prior to deworming shows very high results (95.4%). STH infection was higher (63.6%) in children aged 6–15 years (Table 1).

Table 2 shows that 95.4% out of 88 participants were infected with STH and after deworming, the infection of STH decreased significantly to 53.4% (McNemar test, *p* < 0.001).

Table 3 shows that the highest STH infection was* T. trichiura* (85.2%) and the least common was hookworm (18.2%). The prevalence of* A. lumbricoides* infection was high (71.6%). The intensity of infection was generally light and moderate.

The evaluation six months after deworming showed that the prevalence of infection of each worm species decreased significantly (McNemar test; *p* < 0.001). The prevalence of* T. trichiura* reduced to 39.8%,* A. lumbricoides to* 22.7%, and hookworm to 1.1%. The intensity of infection decreased; there was no heavy and moderate infections except for* T. trichiura* (3 children were still positive with moderate intensity of infection). Classification of each intensity of infection is provided in Supplementary Table 1.

### 3.2. The Nutritional Status before and after Deworming

The data distribution of body weight, height, and IMT of children is not normal (Shapiro-Wilk test *p* < 0.05); therefore, the median is used to count the average. The median weight of children before treatment was 16 kg, height was 113 cm, and BMI was 13.6 kg/m^2^ (Table 4). Six-month evaluation after deworming showed the increase of body weight, height, and BMI. The median weight increased to 18 kg, height to 115 cm, and BMI to 14.4 kg/m^2^. Data on body weight and height were analyzed by plotting body weight/age (1–5 years) and BMI/age (6–15 years) to the WHO chart.

Prior to the deworming, 29 children (33%) were within normal nutritional status, 42 children (47.7%) were underweight, and 17 children (19.3%) were severely underweight (Table 5). Six months after deworming, the nutritional status improved, 66 children were within normal nutritional status (75%), underweight dropped to 22 children (25%), and there were no more children with severe underweight. Wilcoxon test shows a significant difference of nutritional status before and after deworming (*p* < 0.001).

## 4. Discussion

Deworming is an effort in eradicating worms with anthelmintic agents. The effects of deworming, such as the release of 20–30 worms after the treatment, attracted the public to participate in the treatment and education. Education is an important aspect to transform participant's behavior, otherwise reinfection will occur. To prevent reinfection, communities should change their behavior by regularly washing hands before meals and after passing bowels or after contact with soil. However, behavioral changes are difficult to obtain in poor and remote areas especially when the people are low educated. Therefore, WHO recommends performing mass treatment every six months for at least 5 years; consequently, the worms could be controlled without changing the environment and behavior.

### 4.1. The Prevalence of STH before and after Deworming

This study shows a significant decrease in STH prevalence, thus indicating that deworming using albendazole 400 mg for three days was effective in controlling STH. The prevalence of* A. lumbricoides* decreased from 71.6% to 22.7%,* T. trichiura* from 85.2% to 39.8%, and hookworm from 18.2% to 1.1%. Steinmann et al. [9] reported that triple dose of albendazole 400 mg gave higher cure rates compared to single-dose albendazole and single-dose or triple-dose mebendazole. Triple-dose albendazole gave high cure rates for* A. lumbricoides* (96.8%) and hookworm (92%). In* T. trichiura*, the cure rate of triple-dose albendazole was 56.2% and triple-dose mebendazole was 70.7%. Keiser and Utzinger [11] stated that the efficacy of albendazole is higher compared to mebendazole, except for* T. trichiura*.

Aside from decreasing the prevalence, triple dose of albendazole is also able to reduce the intensity of infection which has a role in transmitting the infection through soil contamination. Positive participants will continue to contaminate the environment; thus, if not retreated immediately, the prevalence will increase rapidly. Therefore, the results of this study should be submitted to the local governments to conduct deworming program every six months for a minimum of five consecutive years. In addition, local governments need to improve the environmental condition by providing clean water and latrines, as well as educating people for good hygiene practices, especially the practice of washing hands with soap.

Jia et al. [12] stated that reinfection will occur in 6–12 months after the treatment with albendazole and mebendazole due to failure of behavioral changes, such as defecating indiscriminately and neglecting washing hands at five important times (before eating, after passing stool, before handling babies, after changing diapers, and before preparing food). Appleton et al. [13] reported that within five months there had been a high reinfection of* A. lumbricoides* by 75%,* T. trichiura* by 71%, and hookworm by 48%. Reinfection will return to its baseline prevalence level within 12 months if no retreatment is performed and if retreatment was done twice every six months, the reinfection will be less than 8% [14].

Okoyo et al. [15] stated that despite the occurrence of reinfection, the first three years of deworming reduced the infections with moderate to heavy intensity, as well as the overall prevalence of STH infection. After deworming twice with triple-dose albendazole, the infection with moderate intensity was significantly reduced by 32.7% for STH combination infection, 86.9% for hookworm, 33.9% for* A. lumbricoides*, and* T. trichiura* for 58.4%. Tun et al. [16] performed deworming using triple-dose albendazole for seven years in Myanmar and the results showed a decrease of heavy to moderate intensity infection from 18.5% to less than 7%.

### 4.2. Nutritional Status before and after Deworming

In this study, children who are underweight were 47.7%, normal nutritional status was 33%, and those severely underweight were 19.3%. After deworming, the nutritional status of the children increased; normal was 75%, underweight was 25%, and no severely underweight was found. Wilcoxon test shows a significant difference (*p* < 0.001) between nutritional status of children before and after deworming, which showed that deworming with triple-dose albendazole effectively improved the nutritional status of STH-infected children.

*A. lumbricoides* absorbs nutrients from the intestine, thus causing malabsorption and reducing the cognitive abilities in children.* T. trichiura* adheres in the ascending colon and cecum, which lead to a continuous reflex of defecation; this results in rectal prolapse and chronic diarrhea.* T. trichiura* inflicts a lesion resulting in intestinal bleeding, which may lead to anemia [10, 11, 17, 18]. Hookworms cause chronic intestinal blood loss that result in iron deficiency anemia [10, 18]. Iron deficiency anemia could decrease nutritional intake and gastrointestinal disorders resulting in malnutrition [19].

Deworming could control worms and thus may avoid malabsorption, malnutrition, and anemia and subsequently increase the nutritional status. Therefore, in areas with high risk factors of STH, sampling should be done to determine the prevalence of STH and followed by mass treatment using broad-spectrum anthelmintic agents.

Beside the intestinal worms, malnutrition of children in Perobatang Village is caused by lack of nutrient intake due to the limited daily diet composed of rice and sweet potatoes with no side dishes. Although they live on the coast and most of the residents work as fishermen, people rarely eat fish because fish are for sale; thus, only unsold fish could be consumed. Swine and buffalo are only eaten at certain events, such as wedding ceremony and funeral approximately twice a year. In addition, it is also necessary to provide education to improve nutrition by adding more side dishes (not all fish should be sold), breeding chickens for consumption (for meat and eggs), and farming and cooking meat properly (well done) to avoid tapeworm.

## 5. Conclusion

Deworming with triple-dose albendazole effectively improved the nutritional status of children in Perobatang Village. Before deworming, children with good nutritional status were 33%, underweight were 47.7%, and severely underweight were 19.3%. After deworming, the number of participants with good nutritional status increased to 75%, underweight decreased up to 25%, and severely underweight children were not found. Since the prevalence of STH is high, repeated mass treatment for at least five consecutive years to prevent reinfection is needed.

## Supplementary Material

Supplementary Table 1. Classification of STH infection intensity.

## Figures and Tables

**Table 1:** The prevalence of STH prior to deworming based on age and gender.

Characteristics	Positive STH	95% CI	Negative STH	95% CI
*Age*				
1–5 years	28 (31.8%)	22.1%–41.5%	3 (3.4%)	−0.4–7.2%
6–15 years	56 (63.6%)	53.6%–73.7%	1 (1.2%)	−1–3.5%
Total	84 (95.4%)	91%–99.8%	4 (4.6%)	0.2%–9%
*Gender*				
Male	46 (52.3%)	41.9%–62.7%	2 (2.3%)	−0.8%–5.4%
Female	38 (43.1%)	32.8%–53.5%	2 (2.3%)	−0.8%–5.4%
Total	84 (95.4%)	91%–99.8%	4 (4.6%)	0.2%–9%

CI = confidence interval.

**Table 2:** The prevalence of STH before and after deworming.

Deworming	Positive STH	95% CI	Negative STH	95% CI
Before	84 (95.4%)	91%–99.8%	4 (4.6%)	0.2%–9%
After	47 (53.4%)	43%–63.8%	41 (46.6%)	36.2%–57%

**Table 3:** The prevalence of STH based on intensity of infection before and after deworming.

Infection intensity	*T. trichiura*	*A. lumbricoides*	Hookworm
*Before*			
Heavy	1 (1.1%)	1 (1.1%)	—
Moderate	22 (25%)	30 (34.1%)	—
Light	52 (59.1%)	32 (36.4%)	16 (18.2%)
Total	75 (85.2%)	63 (71.6%)	16 (18.2%)

*After*			
Heavy	—	—	—
Moderate	3 (3.4%)	—	—
Light	32 (36.4%)	20 (22.7%)	1 (1.1%)
Total	35 (39.8%)	22.7%)	1 (1.1%)

**Table 4:** The children anthropometry results before and after deworming.

Deworming		Median	
	Weight (kg)	Height (cm)	BMI (kg/m^2^)
Before	16 (8–39)	113 (72–153)	13.6 (11.6–17.3)
After	18 (9–42)	115 (75–155)	14.4 (12.5–17.3)

**Table 5:** The children nutritional status before and after deworming.

Deworming	Nutritional status					
	Normal	95% CI	Underweight	95% CI	Severely underweight	95% CI
Before	29 (33%)	23.2%–42.8%	42 (47.7%)	37.3%–58.1%	17 (19.3%)	11.1%–27.6%
After	66 (75%)	66%–84%	22 (25%)	16%–34.1%	—	—
