# Supplementary material for: The Effect of Deworming Using Triple-Dose Albendazole on Nutritional Status of Children in Perobatang Village, Southwest Sumba, Indonesia
Source: J Parasitol Res. 2017 Nov 8;2017:5476739. doi: 10.1155/2017/5476739 (PMC5698812; doi:10.1155/2017/5476739)
Supplement: Supplementary file 1 — Supplementary Table 1. Classification of STH infection intensity. [file 5476739.f1.docx]

**Table 1. Intensity of STH Infection**

| **Intensity of Infection** | ***Ascaris sp.*** | ***Trichuris sp.*** | **Hookworms** |
| --- | --- | --- | --- |
| Light | 1–4999 | 1–999 | 1 – 1.999 |
| Moderate | 5000–49.999 | 1000–9999 | 2.000 – 3.999 |
| Heavy | ≥ 50.000 | ≥10.000 | 4.000 |

*Eggs per gram feces
